# The double-edged sword effect of telecommuting on employees’ work engagement: evidence from China during COVID-19

**DOI:** 10.3389/fpsyg.2023.1110108

**Published:** 2023-06-12

**Authors:** Lu Ma, Yunjian Zheng, Ye Wei

**Affiliations:** ^1^School of Management, Guangxi Minzu University, Nanning, China; ^2^School of Management, Minzu University of China, Beijing, China; ^3^School of Economics and Management, Guangxi University of Science and Technology, Liuzhou, China

**Keywords:** telecommuting, work-family conflict, job autonomy, employees’ work engagement, perceived supervisor support, China

## Abstract

**Purpose:**

Drawing on the Job Demands-Resources (JD-R) model, this study aims to explore how telecommuting affects employee’ work engagement, and consider how perceived supervisor support moderates this effect.

**Design/methodology/approach:**

A time-lagged study was conducted on 286 employees from four enterprises in southern China.

**Findings:**

The results showed that telecommuting both decreased work engagement by triggering work–family conflict and enhanced work engagement by increasing job autonomy. In addition, perceived supervisor support enhanced the positive direct effect of telecommuting on job autonomy and the indirect effect on employee’ work engagement, while perceived supervisor support weakened the negative direct effect of telecommuting on work–family conflict and the indirect effect on employee’ work engagement.

**Originality/value:**

This study enrich the literature on telecommuting and employee engagement, and emphasize the importance of perceived supervisor support in this context. Additionally, this study provides some practical implications for companies to adapt and manage telecommuting.

## 1. Introduction

With the rapid development of digital information technology and the outbreak of COVID-19 pandemic, China is experiencing a boom in telecommuting (Cheng and Zhang, 2022). Telecommuting is a mode of working outside the work center with the help of information and communication technology (Nilles, 1975). According to iiMedia Research data, there are more than 300 million people telecommuting in China during the resumption of work in the New Year 2020, which effectively helped enterprises overcome the difficulties of the epidemic and improved employees’ work flexibility and enthusiasm (Cheng and Zhang, 2022). However, some managers are still skeptical about telecommuting. They prefer the traditional management model, which emphasizes strict supervision and control of employees, to virtual management. When companies adopt telecommuting, the biggest concern of managers is that employees’ work engagement and productivity may be greatly reduced due to the lack of direct control (Silva-C et al., 2019). Therefore, as telecommuting becomes increasingly normalized, probing employee attitudes and behaviors at work is an urgent issue for companies to address.

As a key factor in measuring employees’ work attitudes and behaviors, work engagement has been receiving attention from academia and industry (Attridge, 2009; Motyka, 2018; Liu et al., 2021). According to the 2022 China HRM Annual Insight, the annual employee engagement survey has been difficult to meet the needs of enterprises in the digital business environment. Therefore, enterprises are constantly increasing the frequency of employee engagement surveys in order to comprehensively understand the working status of employees and improve management efficiency. At the same time, work engagement, as a comprehensive reflection of employees’ work behavior, emotions, and cognition, also plays an important predictive role in organizational performance and employee turnover intention (Attridge, 2009; Motyka, 2018; Liu et al., 2021). So, how does telecommuting affect employees’ work engagement? Research has generally concluded that telecommuting increases employee’ work engagement (Müller and Niessen, 2019). However, the frequent occurrence of telecommuting buzzwords such as “Working Hours,” “WeChat Sports,” “Parents and Children,” “Miner Partners” in management practices predicts that telecommuting may have a negative impact on employee’ work engagement. Meanwhile, studies by Niu et al. (2021), Athanasiadou and Theriou (2021), and Sardeshmukh et al. (2012) also suggest that telecommuting decreases employees’ work engagement. Based on the above analysis, the impact of telecommuting on employee’ work engagement is likely to have a “double-edged sword” effect. Therefore, this study intends to incorporate both positive and negative effects of telecommuting into the analytical framework, in order to reveal in depth the impact and mechanism of telecommuting on employee’ work engagement.

The Job Demands-Resources (JD-R) model states that job characteristics can be divided into job demands and job resources, and work will have an impact on employees through the loss path caused by job demands and the gain path caused by job resources (Bakker and Demerouti, 2007). Based on this logic, on the one hand, the development of remote interconnection technology and the normalization of epidemic prevention and control will prompt more people to choose telecommuting (Cheng and Zhang, 2022). However, the blurring of work-family boundaries (Nagata et al., 2021), increased unreasonable tasks and enhanced role pressure (Sardeshmukh et al., 2012) caused by telecommuting will occupy and consume employees’ family resources, making them face dual pressure of time and role in the family field, and suffer from work-to-family conflict (Darouei and Pluut, 2021). As a job demand, work–family conflict can further enhance employees’ emotional exhaustion and organizational complaints, reducing their work engagement (Opie and Henn, 2013). On the other hand, telecommuting is characterized by high flexibility, indirect control and self-management (Müller and Niessen, 2019), thus employees have greater job autonomy and decision making power when telecommuting, which will enhance their sense of job autonomy. As a job resource, job autonomy is a necessary condition for employees to achieve self-development and self-enhancement, which will enhance employees’ work enthusiasm and internal motivation, and improve their work engagement. Accordingly, based on the JD-R model, this study suggests that telecommuting may have a dual effect on work engagement through the loss path of work–family conflict and the gain path of job autonomy.

In addition, another focus of this study is to reduce the negative effects of telecommuting and promote its positive effects in order to increase employees’ work engagement. The JD-R model states that job resources can increase employees’ motivation, mitigate the negative effects of work demands on employees, and thus enhance their work engagement (Bakker and Demerouti, 2007). Based on this logic, the supervisor, as the agent of the organization, has high control over work resources, and his or her supportive behavior to employees can promote resource gain, help employees quickly adapt to the telecommuting environment, and alleviate their resource loss (Kirmeyer and Dougherty, 1988). For this reason, the level of supervisor support experienced by employees is likely to affect their work attitude and behavior when telecommuting (Škerlavaj et al., 2014). Thus, this study introduces perceived supervisor support as a moderator of telecommuting influence on employees’ work engagement.

Our study has the following three contributions. First, we confirm that telecommuting has a “double-edged sword” effect on employee’ work engagement, which is useful for managers to understand and manage telecommuting more comprehensively. Second, within the framework of the JD-R model, we reveal that work–family conflict and job autonomy are the mediating mechanisms between telecommuting and work engagement, fully explaining how telecommuting has a “double-edged sword” effect on employee’ work engagement and contributing to the research on telecommuting outcomes. Third, the exploration of telecommuting boundary conditions is facilitated by the introduction of perceived supervisor support.

## 2. Theory and hypotheses

### 2.1. Telecommuting and work engagement

Work engagement refers to a positive and fulfilling mental state shown by employees at work, reflecting their willingness to work (Schaufeli et al., 2002). Studies have shown that work engagement is critical to business development, positively affecting company performance, product quality and customer service, and negatively affecting company costs and employee turnover rates (Attridge, 2009). As the current popular work mode, telecommuting has its own unique characteristics, so how this new way of working will affect employee’ work engagement, it is worth to explore in depth.

Given that telecommuting patterns may alter employees’ work perceptions, emotions, and experiences, and influence their judgments about job demands and resources, thus causing them to exhibit different engagement behaviors (Sardeshmukh et al., 2012). This study draws on the JD-R model in order to theoretically explain the impact of telecommuting on employees (Bakker and Demerouti, 2007). In the framework of the JD-R model, job demands are the negative factors that require sustained individual effort and cause individual resources to lose. Job resources are positive factors that can reduce individual costs, motivate individual growth and development, and promote individual goal achievement (Athanasiadou and Theriou, 2021). According to the JD-R model, the impact of telecommuting on work engagement is likely to be both positive and negative. On the one hand, employees may face occupational isolation, loneliness and blurred work boundaries when telecommuting (Athanasiadou and Theriou, 2021; Niu et al., 2021), and have difficulty in obtaining feedback from superiors and social support (Netemeyer et al., 1996). These job demands can consume employees’ resources such as time, energy and emotions, causing them greater work and psychological stress, reducing their work enthusiasm and organizational identity, and ultimately leading to lower work engagement (Sardeshmukh et al., 2012). On the other hand, telecommuting employees have greater work flexibility and autonomy, and face less political role conflicts (Netemeyer et al., 1996; Müller and Niessen, 2019). These job resources can meet employees’ autonomy needs, enhance their positive emotions and work experience, stimulate their internal motivation, and promote their positive work behaviors, such as work engagement (Sardeshmukh et al., 2012). Therefore, this study attempts to reveal the “double-edged sword” effect of telecommuting on employees’ work engagement from both loss and gain perspectives.

### 2.2. The mediating role of work–family conflict

Work–family conflict is seen as a type of inter role conflict, which includes work hindering the fulfillment of employees’ family obligations and family interfering with employees’ work performance (Netemeyer et al., 1996). It has been shown that the dual pressure of time and role experienced by employees in the family field can easily trigger work–family conflicts, which in turn affect their work attitude and family well-being (Athanasiadou and Theriou, 2021). According to the JD-R model, this study suggests that telecommuting reduces employee’ work engagement through the loss path of work–family conflict.

On the one hand, employees are more likely to face time pressure and role pressure when telecommuting, which can lead to work–family conflict. Specifically, employees face time pressures when they telecommute. First, telecommuting is more focused on work results, and employees will actively invest more time to achieve high performance or passively consume family time to complete unreasonable tasks (Liu et al., 2021). Second, telecommuting places more emphasis on employees being online at all times due to the lack of direct supervision (Silva-C et al., 2019), which blurs the work-family boundary and employees are forced to devote more time to complete tasks (Nagata et al., 2021). However, individual energy and resources are limited, and when employees use too much time and energy at work, the resources used in the family field will decrease (Opie and Henn, 2013). As a result, telecommuting consumes a great deal of employees’ time and energy by increasing workload and job demands, thus hindering the fulfillment of their family roles and further increasing the likelihood of work–family conflict (Darouei and Pluut, 2021). At the same time, employees are prone to role pressure when telecommuting. First, employees who leave the traditional work model need to assume the dual role of commander and executor, rather than just focusing on the execution of tasks (Müller and Niessen, 2019). Second, employees are more likely to suffer from sudden intrusion of work when performing their family roles, thus having to switch back and forth between work and family, thus facing role stress (Elahi et al., 2022). Role stress is an important factor that causes employees to develop negative emotions and behaviors. Based on the spillover effect, employees are likely to spill these negative feelings into the family domain, leading to work–family conflict (Opie and Henn, 2013).

On the other hand, when employees face work–family conflict, work engagement decreases. First, Xanthopoulou et al. (2007) concluded that high work engagement is positively associated with positive mood and healthy psychological states. However, work–family conflict is seen as a job demand that can consume individual resources and lead to emotional exhaustion, resulting in anxiety and dissatisfaction (Müller and Niessen, 2019). These negative emotions spill over into the work domain, which in turn can affect the individual’s performance, such as difficulty concentrating on work or lack of energy to devote to work, ultimately leading to lower work engagement. Second, based on the attribution perspective, employees may attribute the reasons for work–family conflict to the organization, resulting in dissatisfaction and complaints toward the organization, reducing work enthusiasm, and further reducing work engagement (Darouei and Pluut, 2021). It has also been confirmed that work–family conflict can cause employee job burnout, reduce work vitality, and negatively predict employee’ work engagement (Mauno et al., 2007; Opie and Henn, 2013). Thus, the following hypotheses were proposed:

*H1a*: Telecommuting positively affects work-family conflict.

*H1b*: Work-family conflict negatively affects employees’ work engagement.

*H1c*: Telecommuting negatively affects employee’ work engagement through the loss path of work-family conflict.

### 2.3. The mediating role of job autonomy

Job autonomy refers to the extent to which the organization gives employees the right to self-control and self-determination in terms of work methods, organization, time and place (Breaugh, 2016; De Spiegelaere et al., 2016). Job autonomy, as one of the important job characteristics, is a key factor in enhancing employees’ psychological resources and has a positive impact on happiness, job satisfaction and job performance (Prem et al., 2016). According to the JD-R model, this study concludes that telecommuting promotes employees to show high work engagement through the gain path of job autonomy.

On the one hand, telecommuting employees have greater control and decision making power over their work, which in turn enhances job autonomy (Miglioretti et al., 2021). First, telecommuting employees can freely choose their office location and schedule work time, which means that employees have greater autonomy in work arrangements and can complete work tasks according to their own habits and preferences (Sardeshmukh et al., 2012). As a result, employees’ autonomy needs are met, which can reduce work pressure and keep them active at work (De Spiegelaere et al., 2016). Second, telecommuting can help employees avoid excessive interference from colleagues and leaders, which in turn gives them stronger control over their work and allows them to engage in work with ease and freedom (Gajendran and Harrison, 2007). Third, since telecommuting appraisal implements a results-based accountability system, employees have a higher level of control over their work performance. They believe that their own efforts determine work performance, which can enhance employees’ confidence, stimulate their morale, and enhance their autonomy (Golden and Eddleston, 2020). Finally, the reduction of direct supervision in telecommuting weakens employees’ work tension and discomfort, allowing them to try new ways of working freely and boldly, thus enhancing their perception of job autonomy (Harker Martin and MacDonnell, 2012).

On the other hand, a higher degree of job autonomy promotes employees to show high work engagement. First, focusing on self-development and self-enhancement is an important characteristic of employees in the digital economy. As a job resource, job autonomy can provide support for employees to achieve self-development and self-enhancement, stimulate positive emotions and work vitality, make them willing to devote their time and energy to work, and show high work engagement (Mauno et al., 2007). Second, high job autonomy means that employees have some control over their work, while also implying that employees need to be highly responsible for their work (De Spiegelaere et al., 2016). When employees decide and organize their work plans autonomously, their work engagement is enhanced (Sardeshmukh et al., 2012). As a result, employees are able to gain insight into the content, value, and meaning of their work, thus changing their negative perceptions and becoming more dedicated to their work. Finally, employees with high job autonomy will develop the idea that their leaders trust them to perform their job duties, and they are more likely to perceive leadership attention and organizational support (Saragih, 2011). This internal motivation will reinforce employees’ motivation for autonomy and lead them to develop a sense of responsibility. As a result, employees will possess a strong sense of organizational identity and pride and take the initiative to demonstrate dedicated behavior. Prem et al. (2016) found that employees with job autonomy attribute the quality of their work results to their own efforts, and they are more dedicated to achieving positive results. Similarly, Bakker and Demerouti’s (2007) findings suggest that high job autonomy can alleviate employee fatigue and promote employee to exhibit engaged behavior. Therefore, the following hypotheses were proposed:

*H2a*: Telecommuting positively affects job autonomy.

*H2b*: Job autonomy positively affects employee work engagement.

*H2c*: Telecommuting positively affects employee’ work engagement through the gain path of job autonomy.

### 2.4. The moderating role of perceived supervisor support

Perceived supervisor support refers to the extent to which employees perceive that their supervisors value and care about them, depending on the quality of the relationship between supervisors and employees (Eisenberger et al., 1986). As agents of the organization, supervisors are responsible for guiding and evaluating the performance of employees. Their supportive behaviors such as respecting employees’ wishes, valuing their contributions and caring for their well-being at work can meet employees’ work and psychological needs, effectively motivate them to work, help them adapt quickly to the telecommuting environment, and guide them to carry out their work smoothly. Employees who perceive the support of their supervisors will reward the organization with positive work attitudes and behaviors (Babin and Boles, 1996; Yang et al., 2020). Based on the JD-R model, this study argues that the level of supervisor support perceived by employees is likely to influence their work experience in telecommuting.

On the one hand, perceived supervisor support has the potential to reduce work overload, which in turn weakens the negative impact of telecommuting on work–family conflict. Supervisor support, as a way to supplement employee resources, is manifested as the supervisor’s willingness to care about employee’s work and emotional needs and provide them with valuable work resources. For this reason, employees who perceive a high level of supervisor support can feel the care and attention of the organization and their supervisors, and will agree with the decision of the organization to adopt telecommuting. They will learn to manage dual roles to alleviate resource depletion and anxiety caused by telecommuting (Kirmeyer and Dougherty, 1988), effectively balance the relationship between work and family with the help of their supervisors and their own efforts, and reduce work–family conflicts. Conversely, when perceived supervisor support is low, the consumption of resources such as time, energy, and emotions by employees cannot be replenished in a timely manner. This is not conducive to getting employees out of the time and role stress dilemma, but rather reinforces the negative effects of work–family conflict (Wang, 2022). Therefore, the following hypothesis was proposed:

*H3*: Perceived supervisor support moderates the negative relationship between telecommuting and work-family conflict, therefore, such a negative relationship weakens when employees’ perceived supervisor support is higher.

On the other hand, perceived supervisor support has the potential to increase resources, which in turn strengthens the positive impact of telecommuting on job autonomy. Supervisor support, as a supportive behavior, means that supervisors prioritize the achievement of employees’ personal goals and values and provide appropriate assistance and constructive guidance to employees (Eisenberger et al., 1986). Therefore, employees who perceive a high level of supervisor support in telecommuting will make full use of their existing resources, work autonomously with a positive mindset, utilize their own strengths and values (Jose and Mampilly, 2015), and realize value-added resources. Even when facing enormous psychological pressure in autonomous arrangements and decision-making, they will do their best to transform the pressure into motivation to improve themselves and reward the organization. Conversely, when perceived supervisor support is low, employees who have difficulty in obtaining supervisory feedback and approval when telecommuting may have the idea that their work style deviates from the organizational goals, which is not conducive to their work arrangement (Talukder and Galang, 2021), and thus weakens the positive impact of telecommuting on job autonomy. Therefore, the following hypothesis was proposed:

*H4*: Perceived supervisor support moderates the positive relationship between telecommuting and job autonomy, therefore, such a positive relationship enhances when employees’ perceived supervisor support is high.

### 2.5. The moderated-mediation role

Further, based on the above hypotheses, this study proposes a moderated mediation hypothesis, namely that the dual pathway by which telecommuting affects work engagement through work–family conflict and job autonomy is influenced by the level of perceived supervisor support. Specifically, when employees perceive high levels of supervisor support, their work and emotional resources will be supplemented, so they will actively respond to work–family conflicts, enhance job autonomy, and proactively demonstrate high work engagement. On the contrary, when employees perceive low levels of supervisor support, they are prone to resource depletion and negativity, which enhances the negative effects of work–family conflict and hinders the positive effects of job autonomy, resulting in lower work engagement. Thus, the following hypotheses were proposed:

*H5*: Perceived supervisor support negatively moderates the mediation effect of work-family conflict on the relationship between telecommuting and employee’ work engagement.

*H6*: Perceived supervisor support positively moderates the mediation effect of job autonomy on the relationship between telecommuting and employee’ work engagement.

**Figure 1:**
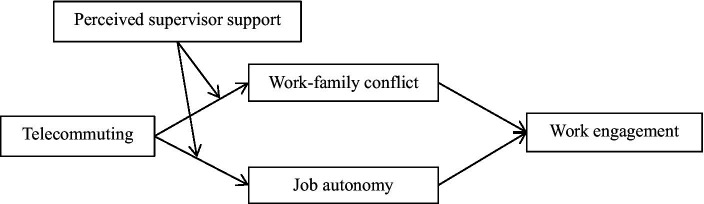
Theoretical model.

To conclude, Figure 1 shows the hypothetical model of this study.

## 3. Materials and methods

### 3.1. Procedure and participants

The participants in this study were from service and technology-based companies in southern China. Four companies implemented telecommuting during the COVID-19 epidemic, and their employees have good telecommuting experience. We used the electronic questionnaire method, and this survey was conducted in two time periods, with a one-month interval before and after. The first survey on demographic information, weekly telecommuting hours, work–family conflict and job autonomy, 380 questionnaires were distributed and 356 were returned, with a return rate of 93.68%. The second survey on perceived supervisor support and work engagement was sent to the sample that submitted the questionnaire, 356 questionnaires were distributed and 322 were returned, with a return rate of 90.45%. In order to screen for subjects with more extensive telecommuting experience, this study was conducted by excluding samples with invalid and weekly telecommuting time as a percentage of total weekly work time less than 10%, and the final number of valid questionnaires obtained was 286, with a sample efficiency of 88.82%. Among the valid sample, 52.8% were male and 47.2% were female; the mean age was 32.23 years (SD = 7.27); the education level is 8% for secondary school and below, 39.2% for college, 39.2% for bachelor’s degree, and 13.6% for master’s degree and above; 25.5% for 2 years of work or less, 64.0% for 3–4 years of work, and 10.5% for 5 years of work or more.

### 3.2. Measurement variables

The questionnaire consisted of six parts: the telecommuting scale, the work–family conflict scale, the job autonomy scale, the perceived supervisor support scale, the work engagement scale, and demographic background information. To ensure the accuracy of the scales originally developed in English, we translated all scales into Chinese following the back-translation procedure in Brislin (1986) study. The questionnaire was measured using the Likert 5-point scale, except for demographic background questions, which were as follows.

#### 3.2.1. Telecommuting

Referring to Spilker and Breaughy (2022), this study used a scale developed by Golden and Veiga (2016), which was self-reported by employees, and which had only one question item, “How long do you telecommute per week?” units in hours. Also, we followed the Golden and Veiga (2016) study, where respondents were also asked about their weekly telecommuting time as a percentage of their total weekly work time, used as a reliability check, and the correlation analysis showed a correlation value of 0.825 between the two measures, indicating no significant difference. Therefore, for the sake of simplicity, we report the number of hours per week that employees telecommuting.

#### 3.2.2. Work–family conflict

We used a 5-item scale developed by Netemeyer et al. (1996). An example item is: “The demands of my work interfere with my home and family life.” The Cronbach’s alpha for this scale was 0.901.

#### 3.2.3. Job autonomy

We used a 7-item scale developed by Kirmeyer and Shirom (1986). An example item is: “I had freedom to decide what to do.” The Cronbach’s alpha for this scale was 0.908.

#### 3.2.4. Perceived supervisor support

We selected and revised the perceived supervisor support scale developed by Eisenberger et al. (1986) and formed a perceived supervisor support load scale with six items. An example item is: “My supervisor cares about my personal goals and value fulfillment.” The Cronbach’s alpha for this scale was 0.876.

#### 3.2.5. Work engagement

We used a 9-item scale containing three dimensions each of vigor, dedication and absorption developed by Schaufeli et al. (2006). Example entries for each of these three dimensions are “At my work, I feel bursting with energy.,” “I am proud of the work that I do.” and “I am immersed in my work.” The Cronbach’s alpha for this scale was 0.865.

#### 3.2.6. Control variables

In this paper, employees’ gender, age, education, and job tenure are used as control variables (Saks, 2006).

## 4. Results

### 4.1. Common method variance analysis

First, the first principal component explained 26.38% of the variance, which did not exceed the recommended value of 40%, in the Harman one-way analysis of variance using SPSS 26.0 in this study. Second, the single-factor model fit was the worst, and the five-factor model fit with the addition of the common method factor (*χ*^2^/df = 1.334, CFI = 0.971, TLI = 0.968, RMSEA = 0.034, SRMR = 0.069) was not better than the four-factor benchmark model. The above results verify that there is no serious common method variance problem in this study.

### 4.2. Confirmatory factor analysis

In this study, confirmatory factor analysis (CFA) was performed on each variable using Mplus 8.1. Because telecommuting intensity is a single-entry measure, it was not placed in the measurement model, which focuses on CFA analysis of work–family conflict, job autonomy, perceived supervisor support, and work engagement. Table 1 shows that the four factor model has the best fitting indicators, indicating good discriminant validity among the variables.

**Table 1:** Results of confirmatory factor analysis.

Measurement models	Factors	*χ* ^2^	df	*χ*^2^/df	CFI	TLI	RMSEA	SRMR
Four-factor model	WF, JA, PS, EE	388.109	318	1.220	0.981	0.979	0.028	0.044
Three-factor model	WF, JA, PS + EE	988.228	321	3.079	0.819	0.802	0.085	0.087
Two-factor model	WF + JA, PS + EE	2141.489	323	6.630	0.507	0.465	0.140	0.190
One-factor model	WF + JA + PS + EE	2449.682	324	7.561	0.424	0.376	0.151	0.158

WF, work–family conflict; JA, job autonomy; PS, perceived supervisor support; EE, work engagement.

### 4.3. Correlation analysis

The descriptive statistics and correlation coefficients for each variable are shown in Table 2. Telecommuting was significantly positively correlated with work–family conflict (*r* = 0.255, *p* < 0.01), significantly positively correlated with job autonomy (*r* = 0.257, *p* < 0.01), work–family conflict was significantly negatively correlated with work engagement (*r* = −0.274, *p* < 0.01), job autonomy was significantly positively correlated with work engagement (*r* = 0.342, *p* < 0.01), perceived supervisor support was significantly negatively related to work–family conflict (*r* = −0.157, *p* < 0.01), and perceived supervisor support was significantly positively related to job autonomy (*r* = 0.356, *p* < 0.01). These results laid the foundation for further testing.

**Table 2:** Descriptive statistical analysis and reliability.

Variables	Mean	SD	1	2	3	4	5
Telecommuting	25.169	9.182	1				
Work–family conflict	2.802	1.038	0.255**	1			
Job autonomy	3.393	0.808	0.257**	0.010	1		
Work engagement	3.614	0.617	0.027	−0.274**	0.342**	1	
Perceived supervisor support	3.461	0.693	0.222**	−0.157**	0.356**	0.339**	1

*N* = 286; **p < 0.01.

### 4.4. Hypothesis testing

**Figure 2:**
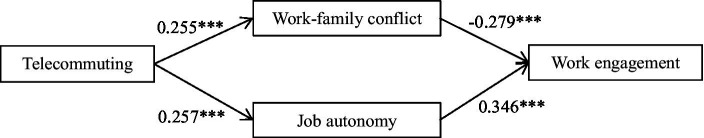
The model’s path coefficients.

First, to test for mediating effects, we performed path analysis using Mplus 8.1 software, the path coefficients of the model are shown in Figure 2, and the results are shown in Table 3. Among the direct effects, telecommuting significantly positively affected work–family conflict (*β* = 0.255, *p* < 0.001), significantly positively affected job autonomy (*β* = 0.257, *p* < 0.001), work–family conflict significantly negatively affected work engagement (*β* = −0.279, *p* < 0.001), and job autonomy significantly positively affected work engagement (*β* = 0.346, *p* < 0.001). Thus, H1a, H1b, H2a, and H2b were verified. Further, in the indirect effect, the test results of 10,000 repeated samples by Bootstrap method showed that the effect value of telecommuting affecting work engagement through work–family conflict was −0.071 with a 95% confidence interval of [−0.008, −0.002] (excluding 0), indicating that the mediating effect of work–family conflict was significantly negative. Thus, H1c was verified. The effect value of telecommuting affecting work engagement through job autonomy was 0.089 with a 95% confidence interval of [0.003, 0.009] (excluding 0), indicating that the mediating effect of job autonomy was significantly positive. Thus, H2c was verified. In addition, the absolute value of the mediating effect of work–family conflict was smaller than the absolute value of the mediating effect of job autonomy (0.071 < 0.089), indicating that the mediating effect of work–family conflict was weaker than the mediating effect of job autonomy.

**Table 3:** Results of mediating effect test.

Effect type	Structural paths	Effect	Boot SE	Boot LLCI	Boot ULCI
Direct effect	TC → WF	0.255***	0.050	0.158	0.350
	TC → JA	0.257***	0.053	0.152	0.361
	WF → EE	−0.279***	0.055	−0.385	−0.173
	JA → EE	0.346***	0.051	0.247	0.446
Indirect effects	TC → WF → EE	−0.071***	0.019	−0.008	−0.002
	TC → JA → EE	0.089***	0.023	0.003	0.009

*N* = 286; ***p < 0.001; Bootstrap sample size = 10,000; TC, telecommuting; WF, work–family conflict; JA, job autonomy; PS, perceived supervisor support; EE, work engagement.

**Figure 3:**
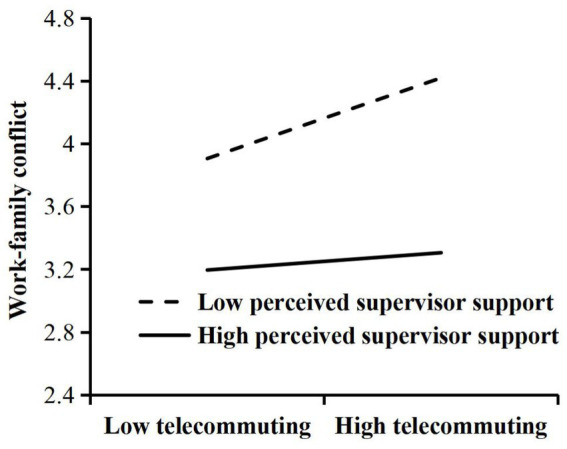
Results of moderating role of perceived supervisor support in the relationship between telecommuting and work–family conflict.

**Figure 4:**
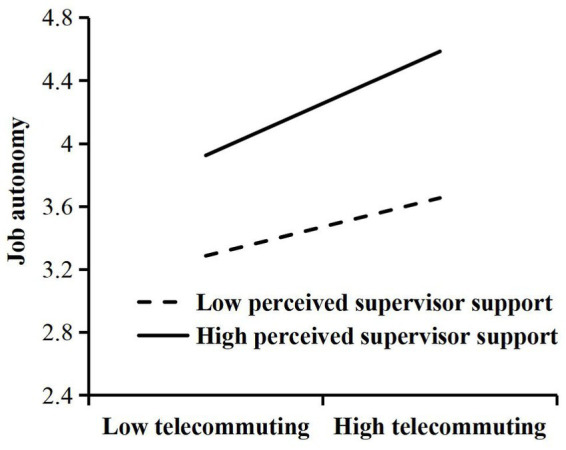
Results of moderating role of perceived supervisor support in the relationship between telecommuting and job autonomy.

Second, we performed a test for moderating effects, and the results are shown in Table 4. H3 argued that the perceived supervisor support moderated the relationship between telecommuting and work–family conflict. From Model 2, the interaction term of telecommuting and perceived supervisor support had a significant effect on work–family conflict (*β* = −0.022, *p* < 0.01), indicating that perceived supervisor support moderated the relationship between telecommuting and work–family conflict, and this moderating effect is shown in Figure 3, where high perceived supervisor support can weaken the negative effect of telecommuting on work–family conflict. Therefore, H3 was verified. H4 argued that the perceived supervisor support moderated the relationship between telecommuting and job autonomy. From model 4, the interaction term of telecommuting and perceived supervisor support had a significant effect on job autonomy (*β* = 0.016, *p* < 0.05), indicating that perceived supervisor support moderated the relationship between telecommuting and job autonomy, and this moderating effect is shown in Figure 4, where high perceived supervisor support can enhance the positive effect of telecommuting on job autonomy. Therefore, H4 was verified.

**Table 4:** Results of moderating effect test.

Variables	Work–family conflict		Job autonomy	
	Model 1	Model 2	Model 3	Model 4
Intercept	3.444***	3.457***	2.975***	2.966***
Gender	−0.042	−0.021	0.040	0.025
Age	−0.011	−0.010	0.006	0.005
Education	−0.042	−0.066	0.018	0.036
Job tenure	−0.065	−0.056	0.054	0.047
Telecommuting	0.034***	0.028***	0.016**	0.020***
Perceived supervisor support	−0.339***	−0.358***	0.368***	0.382***
Telecommuting × Perceived supervisor support		−0.022**		0.016*
*R* ^2^	0.126	0.148	0.170	0.188
*∆R* ^2^		0.022		0.018
*F*	6.551***	6.712***	9.284***	8.970***

*N* = 286; *p < 0.05, **p < 0.01, ***p < 0.001.

Finally, we conducted a test of moderated mediation effect. In this study, Bootstrap method was used to test H5 and H6, and the results are shown in Table 5. From Table 5, the effect of telecommuting on work engagement through work–family conflict presents different results depending on the level of perceived supervisor support. Specifically, in the condition of low perceived supervisor support (-1SD), the indirect effect of work–family conflict was significant [*β* = −0.007, 95% CI = (−0.012, −0.004)]; in the condition of high perceived supervisor support (+1SD), the indirect effect of work–family conflict was not significant [*β* = −0.002, 95% CI = (−0.005, 0.001)]. The difference between the two effect coefficients was significant [Δ*β* = 0.005, 95% CI = (0.002, 0.010)]. Thus, the perceived supervisor support moderated the mediating role of work–family conflict between telecommuting and work engagement, and H5 was verified. In addition, the effect of telecommuting on work engagement through job autonomy presents different results depending on the level of perceived supervisor support. Specifically, in the condition of low perceived supervisor support (-1SD), the indirect effect of job autonomy was not significant [*β* = 0.002, 95% CI = (0.000, 0.005)]; in the condition of high perceived supervisor support (+1SD), the indirect effect of job autonomy was significant [*β* = 0.008, 95% CI = (0.004, 0.014)]. The difference between the two effect coefficients was significant [Δ*β* = 0.006, 95% CI = (0.002, 0.011)]. Thus, perceived supervisor support moderated the mediating role of job autonomy between telecommuting and work engagement, and H6 was verified.

**Table 5:** Results of moderated mediating effect test.

Moderator	Perceived supervisor support	Effect	Boot SE	Boot LLCI	Boot ULCI
Work–family conflict	High (+1SD)	−0.002	0.001	−0.005	0.001
	Low (-1SD)	−0.007	0.002	−0.012	−0.004
	Effect difference	0.005	0.002	0.002	0.010
Job autonomy	High (+1SD)	0.008	0.003	0.004	0.014
	Low (-1SD)	0.002	0.001	0.000	0.005
	Effect difference	0.006	0.002	0.002	0.011

## 5. Discussion

In this study, we investigated how telecommuting affects employee’ work engagement during the COVID-19 in China. Applying the JD-R model, we examined the “double-edged sword” effect of telecommuting on employee’ work engagement. In addition, we found that perceived supervisor support is an important boundary condition to reduce the negative effects of telecommuting and promote its positive effects.

### 5.1. Theoretical implications

This study makes three main theoretical contributions to the literature. First, this study deepens the understanding of the effectiveness of telecommuting and expands the in-depth analysis of the effects of telecommuting on employee’ work engagement. Previous research on telecommuting has mostly been conducted in a Western context (Golden and Veiga, 2016; Silva-C et al., 2019; Golden and Eddleston, 2020), leading to limited generalizability. Driven by the digital wave sweeping the world and the outbreak of the COVID-19 pandemic, telecommuting has emerged in China at an unstoppable pace. Therefore, it is necessary to understand the role of telecommuting in the Chinese context to strengthen the literature and external validity. In addition, previous studies have focused on the effects of telecommuting on employee health (Niu et al., 2021; Cheng and Zhang, 2022), satisfaction (Virick et al., 2009; Golden and Veiga, 2016), and job performance (Golden and Gajendran, 2018; Liu et al., 2021). However, studies on work engagement are scarce and their findings are variable, probably due to differences in culture and research perspectives (Sardeshmukh et al., 2012; Masuda et al., 2017; Nagata et al., 2021; Wang et al., 2023). By examining the impact of telecommuting on employee work engagement in the Chinese context, this study expands the research context of telecommuting, enriches the research on the consequences of telecommuting, and responds positively to Miglioretti et al.’s (2021) suggestion that “academia and industry should pay close attention to the quality of employee telecommuting.”

Second, within the framework of the JD-R model, this study constructs a dual-path model in which telecommuting affects work engagement by triggering work–family conflict and enhancing job autonomy, uncovering the “black box” between them. Specifically, in the loss path, studies have focused on the time pressure, role ambiguity, and role conflict (Sardeshmukh et al., 2012), but exploring the role of work–family conflict as a mediator between telecommuting and work engagement provides insight into the resource depletion dilemma faced by employees in the family field and responds to the suggestion of Sardeshmukh et al. (2012) and Nagata et al. (2021) to “incorporate work–family conflict into telecommuting impact studies.” In the gain path, consistent with Parent-Lamarche’s (2022) findings, job autonomy contributes positively to the relationship between telecommuting and work engagement. This is because job autonomy, as a job resource for employees, meets their self-development and self-improvement needs, and is an important tool for motivating employees. In addition, this study compared differences in the mediating effects of work–family conflict and job autonomy, and found that the positive effect of the gain path is greater than the negative effect of the loss path, which means that employees pay more attention to job autonomy when telecommuting. The research results respond to the call of Boell et al. (2016) to integrate and analyze the positive and negative effects of telecommuting.

Third, drawing on the JD-R model, this study reveals the boundary of the “double-edged sword” path of telecommuting, that is, the moderating effect of supervisor support, and thus clarifies the contexts that are more conducive to the improvement of telecommuting employees’ work engagement. In existing studies, most have confirmed the positive effect of perceived supervisor support, which can reduce employees’ turnover intention (Zhang et al., 2020) and positively affect their proactive adjustment behaviors (Zhang et al., 2014), work engagement (Jose and Mampilly, 2015), and idea implementation (Škerlavaj et al., 2014). However, a few highlight the “dark side” of perceived supervisor support, in that it can lead to unethical behavior and that this effect can be accentuated by employees’ Machiavellianism (Li et al., 2022). This study explores the role of perceived supervisor support in the context of telecommuting in China, and the results indicate that the level of perceived supervisor support plays an important role in the loss and gain paths of telecommuting to work engagement. A high perceived supervisor support reinforces the positive effects of telecommuting and motivates employees to demonstrate high levels of work engagement, while a low perceived supervisor support amplifies the negative effects of telecommuting and reduces employee’ work engagement. These results confirm the positive role of perceived supervisor support, which is related to the lack of work resources in telecommuting and the characteristics of collectivism culture in China. Meanwhile, the above results also support the idea that “perceived supervisor support is a key element in promoting resource gain and buffering work demand overload” (Jose and Mampilly, 2015), confirm the finding that “the quality of the relationship between employees and supervisors affects work engagement” (Talukder and Galang, 2021; Mori et al., 2022), enrich the study of the moderating mechanism in the effect of telecommuting.

### 5.2. Practical implications

This study makes two key practical contributions to the literature. First, in view of the “double-edged sword” effect of telecommuting on employee’ work engagement, managers need to be aware of both the job autonomy it brings and the work–family conflict it creates. On the one hand, supervisors can conduct training on coping with loneliness, stress and conflict to help employees learn to balance work and family, so that they can maintain a high level of work engagement at home, thereby reducing the negative impact of work–family conflict. On the other hand, job autonomy, as a job resource, is an important factor in alleviating employee resource depletion, increasing employee engagement and improving organizational trust. Supervisors need to redesign their work models, adopt trust management and give employees appropriate voice and decision-making power to enhance their job autonomy and thus increase their work engagement.

Second, this study found that the perceived supervisor support is an important boundary for improving employee’ work engagement in telecommuting. Therefore, enterprises, supervisors and employees should pay attention to the importance of perceived supervisor support. First, enterprises should focus on developing supervisors’ awareness of support, incorporating their supportive behavior into the assessment system, in order to strengthen their understanding of the importance of implementing supportive behavior and encourage them to demonstrate supportive behavior. Second, supervisors should learn to establish effective two-way communication mechanisms with employees, identify their needs and provide necessary help and support in a timely manner, thereby creating a supportive organizational climate and improving employees’ perception of supervisor support. Finally, employees need to improve their ability to perceive support from their supervisors, proactively maintain contact with them and timely access resources such as tools and emotions in order to cope with the difficulties and challenges, ultimately realizing their value in the organization.

### 5.3. Limitations and future research

This study has several limitations worth discussing. First, all of the measurements in this study used employee self-assessments, which may affect the accuracy of the data and suffer from common method bias, and future studies could use multiple measures and collect data from different subject sources to improve the causal relationships between these variables.

Second, in terms of control variables, this study only controlled demographic variables that may have an impact on work engagement, while ignoring telecommuting. Previous research has shown that work experience, job position, and industry may have an impact on telecommuting (Masuda et al., 2017; Wang et al., 2023). Therefore, future research needs to pay attention to these variables.

Third, this study was conducted in the context of the COVID-19 in China with a study sample of Chinese telecommuting employees, which affected the external validity of the findings. Therefore, the theoretical model could be tested with samples from other countries in the future.

Finally, this study only analyzed the mediating effects of job autonomy and work–family conflict, and future research can further explore the chain mediating effect between the two. In addition, this study only examined the moderating effect of perceived supervisor support. In the relationship between telecommuting and employees’ work engagement, leadership style (such as electronic leadership, transformational leadership), individual characteristics (such as self-leadership, self-efficacy, gender differences), and social support (such as colleague support behavior) are also the moderating variables that we should explore.

## 6. Conclusion

This study explored the potential relationship between telecommuting and employee’ work engagement using a sample of Chinese telecommuting employees during the COVID-19 pandemic. The results showed that telecommuting not only triggered work–family conflict and thus decreased work engagement, but also enhanced employees’ job autonomy and thus increased work engagement, showing a “double-edged sword” effect. In addition, perceived supervisor support moderated these relationships, and high levels of perceived supervisor support could reduce work–family conflict, increase job autonomy, and promote employees to show high work engagement. This study was conducted within the framework of the JD-R model, and these findings expanded our understanding of telecommuting outcomes during the COVID-19 pandemic, helped supervisors better improve the work engagement of telecommuting employees, and also urged future research to focus more on this field.

## Data availability statement

The raw data supporting the conclusions of this article will be made available by the authors, without undue reservation.

## Author contributions

LM, YZ, and YW: research design. YZ: data collection and data analysis. YZ: writing of the original draft. LM, YZ, and YW: revising the article. All authors contributed to the article and approved the submitted version.

## Funding

This study was supported by the National Social Science Foundation of China (Grant No. 20XGL014).

## Conflict of interest

The authors declare that the research was conducted in the absence of any commercial or financial relationships that could be construed as a potential conflict of interest.

## Publisher’s note

All claims expressed in this article are solely those of the authors and do not necessarily represent those of their affiliated organizations, or those of the publisher, the editors and the reviewers. Any product that may be evaluated in this article, or claim that may be made by its manufacturer, is not guaranteed or endorsed by the publisher.
